# RALBP1 in Oxidative Stress and Mitochondrial Dysfunction in Alzheimer’s Disease

**DOI:** 10.3390/cells10113113

**Published:** 2021-11-10

**Authors:** Sanjay Awasthi, Ashly Hindle, Neha A. Sawant, Mathew George, Murali Vijayan, Sudhir Kshirsagar, Hallie Morton, Lloyd E. Bunquin, Philip T. Palade, J. Josh Lawrence, Hafiz Khan, Chhanda Bose, P. Hemachandra Reddy, Sharda P. Singh

**Affiliations:** 1 Department of Internal Medicine, Texas Tech University Health Sciences Center, Lubbock, TX 79430, USA; sanjay.awasthi@ttuhsc.edu (S.A.); ashly.hindle@ttuhsc.edu (A.H.); neha.sawant@ttuhsc.edu (N.A.S.); mathewkgeorge16@gmail.com (M.G.); murali.vijayan@ttuhsc.edu (M.V.); Sudhir.kshirsagar@ttuhsc.edu (S.K.); hallie.morton@ttuhsc.edu (H.M.); Lloyd.e.bunquin@ttuhsc.edu (L.E.B.); chhanda.bose@ttuhsc.edu (C.B.); 2 Department of Pharmacology and Toxicology, University of Arkansas for Medical Sciences, Little Rock, AR 72205, USA; ppalade@uams.edu; 3 Department of Pharmacology and Neuroscience and Garrison Institute on Aging, Texas Tech University Health Sciences Center, Lubbock, TX 79430, USA; John.lawrence@ttuhsc.edu; 4 Department of Public Health, Graduate School of Biomedical Sciences, Texas Tech University Health Sciences Center, Lubbock, TX 79430, USA; Hafiz.khan@ttuhsc.edu; 5 Department of Neurology, Texas Tech University Health Sciences Center, Lubbock, TX 79430, USA; 6 Department of Speech, Language, and Hearing Sciences, Texas Tech University Health Sciences Center, Lubbock, TX 79430, USA

**Keywords:** Alzheimer’s disease, mitochondria, mitophagy, mitochondrial biogenesis, synaptic proteins

## Abstract

The purpose of our study is to understand the role of the *RALBP1* gene in oxidative stress (OS), mitochondrial dysfunction and cognition in Alzheimer’s disease (AD) pathogenesis. The *RALPB1* gene encodes the 76 kDa protein RLIP76 (Rlip). Rlip functions as a stress-responsive/protective transporter of glutathione conjugates (GS-E) and xenobiotic toxins. We hypothesized that Rlip may play an important role in maintaining cognitive function. The aim of this study is to determine whether Rlip deficiency in mice is associated with AD-like cognitive and mitochondrial dysfunction. Brain tissue obtained from cohorts of wildtype (WT) and Rlip^+/−^ mice were analyzed for OS markers, expression of genes that regulate mitochondrial fission/fusion, and synaptic integrity. We also examined mitochondrial ultrastructure in brains obtained from these mice and further analyzed the impact of Rlip deficiency on gene networks of AD, aging, stress response, mitochondrial function, and CREB signaling. Our studies revealed a significant increase in the levels of OS markers and alterations in the expression of genes and proteins involved in mitochondrial biogenesis, dynamics and synapses in brain tissues from these mice. Furthermore, we compared the cognitive function of WT and Rlip^+/−^ mice. Behavioral, basic motor and sensory function tests in Rlip^+/−^ mice revealed cognitive decline, similar to AD. Gene network analysis indicated dysregulation of stress-activated gene expression, mitochondrial function and CREB signaling genes in the Rlip^+/−^ mouse brain. Our results suggest that Rlip deficiency-associated increases in OS and mitochondrial dysfunction could contribute to the development or progression of OS-related AD processes.

## 1. Introduction

Alzheimer’s disease (AD), the most common form of dementia, is irreversible, progressive and heterogeneous, having several etiologies and pathological processes that evolve as it progresses from mild cognitive impairment to dementia [1]. AD is the 6th leading cause of death and is the only disease among the 10 leading causes of death in the United States that cannot be cured, prevented or slowed [2,3]. AD occurs in two forms: early onset familial and late-onset sporadic. Synaptic damage and loss correlate strongly with cognitive dysfunction in patients with AD [4,5,6,7]. Several years of research have revealed multiple cellular changes that are involved in disease progression, including microRNA deregulation, synaptic damage, the proliferation of astrocytes and glia, oxidative stress (OS), mitochondrial structural and functional abnormalities, hormonal imbalance, formation and accumulation of Aβ and p-Tau, and neuronal loss [8,9,10,11,12,13]. 

In the brains of AD patients, increased levels of free radicals and OS have been extensively reported [14,15,16,17,18,19], and this is also true of other neurodegenerative diseases [20,21,22]. It has been frequently theorized that reactive oxygen species (ROS) arise from mitochondrial dysfunction, resulting in a positive feedback loop that is thought to further damage mitochondria, cause DNA damage, and exacerbate neurodegeneration [23,24]. Chief among the detrimental ROS produced is 4-hydroxynonenal (4HNE), a toxic peroxidation product of polyunsaturated fatty acids (PUFAs) [25]. Environmental toxins, heavy metals, cigarette smoke, chronic inflammation and most other risk factors for AD have in common the ability to initiate or promote lipid peroxidation (LPO) [14,26]. Superoxide anions (O_2_^−●^, the unpaired ‘radical’ electron shown as ^●^) react with PUFAs to yield toxic free radicals (i.e., OH^●^, O_2_^−●^, etc.) and electrophilic lipid metabolites (LOO^●^). Given its high lipid composition, it is not surprising that LPO is highly toxic to brain tissue and is a hallmark of late-stage AD and other neurodegenerative diseases [26]. 

The development of drugs that activate the expression of glutathione (GSH)-linked antioxidant enzymes (GLAE), which metabolize LOO^●^ and electrophilic lipids, is critically important because impaired GLAE activation is a key pathogenic mechanism of AD [27,28]. GLAE expression is upregulated by Nrf2, a transcription factor stabilized by the reaction of electrophilic lipids with its binding partner, KEAP1 [29]. Nrf2 binds an antioxidant response element (ARE) to induce transcription of GLAEs [29]. The stress-responsive transcription factor CREB (cAMP regulatory element binding protein), along with its co-activators CBP (CREB-binding protein) and EP300, as well as HSF1, the master TF for heat-shock proteins, and its co-activator p53 interact with and modulate Nrf2 functions [30,31,32]. 

Mercapturic acid pathway transporters remove mercapturic acid precursor GSH-electrophile conjugates (GS-E) from cells [33,34,35]. *RALBP1* encodes RLIP76 (referred to here as Rlip), a 76 kDa splice variant protein that binds the clathrin-dependent endocytosis (CDE) complex through the clathrin adaptor protein AP2 [36,37]. Rlip possesses ATPase and GS-E transport activities that are coupled with the internalization of peptide hormone/receptor complexes by CDE [36,37], and it is present in intracellular tyrosine kinase signaling complexes that also contain Grb2/Nck and Shc [36,38,39,40]. Importantly, Rlip has a key role in the efflux of glutathione conjugates of 4HNE (GS-HNE) from the cell, decreasing oxidative stress and preventing the feedback inhibition of GSTA4, an important glutathione utilization enzyme. We have found that Rlip knockout mice have impaired CDE, structurally abnormal mitochondria and high levels of OS [41,42,43]. In response to high OS, it would be expected that Nrf2 and GLAE would be increased and that GSH would be low due to increased consumption by GSH-utilizing enzymes. Surprisingly, in the findings presented here, although Nrf2 expression was increased, glutathione peroxidase (GPx) activity, aldose reductase (AR) activity, and GSH conjugation of 4HNE (forming GS-HNE) by GSTA4 were lower in heterozygous Rlip knockout (Rlip^+/−^) mice than in wildtype (WT, genotype Rlip^+/+^) mice, perhaps due to a deficiency in the activation of GLAE genes as a consequence of reduced Rlip. In the case of GSTA4, the reduced conjugation activity may also be a result of feedback inhibition by accumulated GS-HNE in Rlip^+/−^ cells, since Rlip is responsible for roughly 80% of GS-HNE efflux from cells [44].

We recently reported that Rlip depletion by antisense exerted genome-wide alterations on gene promoter CpG island methylation [42]. RNA sequencing of Rlip-depleted mice, in combination with pathway analysis, confirmed that among the top differentially expressed canonical pathways were those involved with OS response and endocytosis, both functions which are critical for proper neuronal functioning. Nrf2 was among the top differentially expressed genes by RNA-Seq studies, and the Nrf2 gene promoter was among the top differentially methylated regions (DMR) [42]. These studies suggest a link between Nrf2 and the curious finding that many of the top differentially expressed pathways were neuronal (CREB signaling in neurons, axonal guidance and long-term potentiation). Thus, it is possible that Rlip depletion may not only *directly* exacerbate oxidative damage, with its consequent neurodegeneration and cognitive deterioration, by preventing the efflux of 4HNE, but also *indirectly* exacerbate neurodegeneration via effects on the regulation of OS response pathways and the signaling networks which induce anti-oxidant enzymes, including the CREB and Nrf2 networks.

In this work we chose to focus on Rlip^+/−^ mice rather than Rlip^−/−^ mice for two reasons. Firstly, Rlip^−/−^ mice are smaller than WT littermates, which would likely affect performance on behavioral tests. Rlip^+/−^ mice, by contrast, have no readily apparent differences from WT mice. Secondly, the Rlip^+/−^ heterozygous genotype represents a degree of expression change that is much more clinically plausible than that modeled by Rlip^−/−^ mice. It is highly unlikely that Rlip expression would be fully lost in the brains of AD patients, as would be modeled by Rlip^−/−^ mice. For this reason, we also chose not to perform molecular studies on brains taken from Rlip^−/−^ mice, although aberrations in mitochondrial, synaptic, and OS pathways are highly likely to exist in Rlip^−/−^ neurons. 

In the present study, we report for the first time that Rlip^+/−^ mice had some neurocognitive deficits that resemble AD and that Nrf2-regulated antioxidant enzyme activities were reduced in the brains of these mice. Thus, Rlip^+/−^ mice represent a unique tool for studying the progression of AD. We further explored the novel possibility that the mitochondrial biogenesis, mitochondrial dynamics, and synaptic proteins which are typically aberrantly expressed in AD were also altered in Rlip^+/−^ mice [10]. PGC1a, Nrf1, Nrf2, and TFAM were assessed as markers of mitochondrial biogenesis [45,46]. To study mitochondrial dynamics, we examined the expression of the mitochondrial fission proteins Drp1 and Fis1 and the mitochondrial fusion proteins Mfn1, Mfn2, and Opa1 [45,47]. The expression of synaptophysin and PSD95 was used to make inferences regarding synaptic health [45,48]. In this work, we have examined whether the heterozygous loss of Rlip alters the expression of these proteins in the hippocampus and the cerebral cortex, brain regions which are impacted in AD patients. These studies will offer novel insights into the regulation of OS defenses in AD, lead to new strategies for the treatment of AD, and offer insights into the roles of OS and mitochondrial dysfunction in the etiology of AD and possibly other neurodegenerative diseases. 

## 2. Methods

### 2.1. Euthanasia and Necropsy

Mice were used under Protocol #18015, approved by the TTUHSC Institutional Animal Care and Use Committee and in accordance with the Declaration of Helsinki. Brains were extracted, hemisected, and weighed. One hemisphere was fixed in formalin while the other hemisphere was snap frozen at −80 °C or homogenized fresh for molecular and biochemical analysis.

### 2.2. Behavioral Testing

#### 2.2.1. Open Field Test

The Open Field Test evaluates not only locomotion but also anxiety, because mice that are anxious will tend to avoid the center of the field. Each mouse was acclimated to the testing room for 60 min. They were then placed into the center of a dimly lit (20–30 lux) Open Field Test apparatus (44 cm × 44 cm × 30 cm) and allowed to explore the entirety of the box for 10 min. In order to remove olfactory cues of urine from the surface of the apparatus, the apparatus was cleaned thoroughly with 30% ethanol between experiments. The mouse’s movement in the Open Field arena was tracked using the ANY-maze automated tracking system [49,50,51,52].

#### 2.2.2. Morris Water Maze (MWM)

The Morris Water Maze test is specifically utilized for the measurement of hippocampal function and examining brain deficits in diseased mice [53]. The test uses a tank filled with milky water to motivate the animal to escape onto the hidden platform just below the water’s surface. If the mouse reaches the platform before the allotted time, the test is terminated. Mice were trained and tested in a large, circular, galvanized steel pool (160 cm in diameter, 62 cm high, filled with 26 ± 1 °C water to a height of 24 cm) to find a hidden platform (10 cm in diameter, located 1.5 cm below the surface of the water). 10-month-old mice (5 WT and 5 Rlip^+/−^) were placed in a galvanized circular tank with cloudy water. The test was run for a maximum time of 60 s. The ANY-maze automated tracking system analyzed latency to escape and time spent in the target quadrant.

#### 2.2.3. Rotarod Test

Motor coordination and learning were assessed using the Rotarod Test. Mice were placed on an Ajanta three-compartment Rotarod, with a horizontal rod rotating on its long axis that required mice to walk forward to avoid falling off of the rod [54]. The mice were placed on the rod rotating at 18 RPM, timed, and given a maximum running time of five minutes. 

### 2.3. Quantification of mRNA Expression Using Real-Time PCR

Quantitative RT-PCR (qRT-PCR) was performed on an Applied Biosystems 7900HT Fast Real-Time PCR System (Thermo Fisher Scientific, Waltham, MA, USA) using SYBR Green Master Mix. 1 μg of total RNA from the brain was used to synthesize cDNA using M-MLV reverse transcriptase (Thermo Fisher). Total RNA was isolated from mouse brain using TRIzol Reagent (Thermo Fisher). Primers for qRT-PCR were designed using Primer Express Software version 3.0.1 (Thermo Fisher) for the housekeeping and test genes. Primer sequences are given in Table 1. qRT-PCR was performed in replicate on a 7900HT Fast Real-Time PCR System with PowerUp SYBR Green Master Mix (Thermo Fisher) in a total reaction volume of 20 µL containing 0.3 µM gene-specific primers. The cycling protocol was an initial denaturation at 95 °C, followed by 40 cycles of denaturation at 95 °C and annealing/extension at 60 °C. The beta-actin transcript was used as a reference for the normalization of mRNA levels. Gene expression levels were normalized for each individual animal by the ∆∆Ct method [55].

### 2.4. Western Blot Analysis

Western blot analysis was performed using protein lysates prepared from Rlip^+/−^ and WT mice. To normalize the expression levels of mitochondrial biogenesis, mitochondrial dynamics, and synaptic proteins, we have used beta-actin as an internal control. Details of antibody dilutions are given in Table 2. After treatment, cells were lysed in 50 μL cold RIPA lysis buffer (MilliporeSigma, Burlington, MA, USA) for 60 min on ice (vortexing every 15 min) and centrifuged at 12,000× *g* for 11 min. After centrifugation, the supernatant was collected, and protein concentration was measured. 40 μg protein preparation in LDS buffer was loaded, electrophoretically separated by SDS-PAGE gels (10%) and transferred to a polyvinylidene difluoride (PVDF) membrane (cat# 10026933, Bio-Rad Laboratories, Hercules, CA, USA). Blocking was performed by adding 5% BSA in TBST for 60 min at room temperature on the shaker. After washing 2 times, primary antibody was added to the membranes overnight at 4 °C. The membrane was washed 3 times with TBST and incubated with HRP (horseradish peroxidase)-labeled secondary antibodies for 1 h at room temperature. Proteins were detected with chemiluminescence substrate (ECL, Thermo Fisher), and the band exposures were kept within the linear range. Bands from immunoblots were quantified using densitometry on a Kodak Scanner (ID Image Analysis Software, Kodak Digital Science, Kennesaw, GA, USA). ImageJ software version 1.46r was used to quantify band intensity and determine statistical significance. 

### 2.5. Immunofluorescence Analysis and Quantification

Immunofluorescence analysis was performed using coronal hippocampal sections from WT and Rlip^+/−^ mice on mitochondrial dynamics, mitochondrial biogenesis, and synaptic proteins. Antibody dilutions of primary and secondary antibodies are given in Table 3. The sections (around 3–4 µm) were allowed to reach room temperature (~30 min) and rinsed at room temperature in phosphate buffered saline (PBS) for 5 min. The sections were then fixed in freshly prepared 4% paraformaldehyde in PBS for 10 min, washed with PBS and permeabilized with 0.1% Triton-X100 in PBS. They were blocked with a 1% blocking solution for 1 h at room temperature. All sections were incubated overnight with primary antibodies. After incubation, the sections were washed three times with PBS for 5 min each. The sections were incubated with a secondary antibody conjugated with Alexa Fluor 488 (Invitrogen) for 1 h at room temperature. The sections were washed three times with PBS. After washing the sections, the coverslips were mounted on glass with Prolong Diamond antifade mounting medium (Thermo Fisher) with DAPI for the identification of nuclei. Photographs were taken with a Nikon Eclipse E600 epifluorescence microscope system (Nikon Corporation, Tokyo, Japan). To quantify the immunoreactivity of mitochondrial and synaptic antibodies for each treatment, 10–15 photographs were taken at 40× magnification as described in our previous publications [56,57]. 

### 2.6. Aldose Reductase Assay

Brain lysates were prepared in G1 buffer (20 mM potassium phosphate, pH 7.0; 1.4 mM BME; and 2 mM EDTA), and AR activity was assayed according to the method described previously [58]. Briefly, the assay mixture contained 50 μM potassium phosphate buffer (pH 6.2), 0.4 mM lithium sulfate, 5 μM 2-mercaptoethanol, 10 μM DL-glyceraldehyde, 0.1 μM NADPH, and enzyme preparation (10 µg/assay). The assay mixture was incubated at 37 °C and initiated by the addition of NADPH at 37 °C. The change in the absorbance at 340 nm due to NADPH oxidation was measured using a SpectraMax Plus (Molecular Devices, San Jose, CA, USA) spectrophotometer.

### 2.7. Glutathione Conjugation of 4-Hydroxynonenal

4-HNE conjugating activity with glutathione was determined by the method of Alin, described previously [59,60,61]. Briefly, assay mixture contained 100 mM Kpi buffer (pH 6.5), 0.5 mM GSH and 0.1 mM 4-HNE. The blank was without protein, but with all other components, including 4-HNE (added at time 0), included, since the rate of non-enzymatic reaction of 4-HNE with GSH is relatively high. Measurement was started immediately after adding 4-HNE, since the reaction rate is linear with time for a short time only. The production of glutathione-conjugated 4-HNE was measured spectrophotometrically in cell lysates using the absorbance at 224 nm. The rate of product formation is a function of the enzymatic activity of GSTA4.

### 2.8. Glutathione Peroxidase Assay with Cumene Hydroperoxide

Intracellular glutathione peroxidase enzyme activity was determined in brain homogenates using the Glutathione Peroxidase Assay Kit from Abcam (Colorimetric; ab102530) [62]. Briefly, tissues were homogenized in a hypotonic lysis buffer and sonicated. Glutathione peroxidase (GPx) oxidizes GSH to produce GSSG as part of the reaction in which it reduces cumene hydroperoxide. Glutathione reductase (GR) then reduces the GSSG to produce GSH, and in the same reaction, consumes NADPH. The decrease in NADPH (measured at OD = 340 nm) is proportional to GPx activity.

### 2.9. Electron Microscopy

To determine the effects of Rlip deficiency on mitochondrial number and size, we performed transmission electron microscopy in hippocampal and cortical sections of 10-month-old Rlip^+/−^ mice relative to age-matched WT mice. Animals were perfused using the standard perfusion method—after successful perfusion, skin was removed on top of the head, and the brain was taken out and post-fixed for 2–3 h and/or indefinitely before cutting the hippocampal and cortical sections for transmission electron microscopy. 1 mm × 1 mm thickness fixed sections from hippocampi and cortices were used for further analysis. Cut sections were stained for 5 min in lead citrate. They were rinsed and post-stained for 30 min in uranyl acetate and then were rinsed again and dried. Electron microscopy was performed at 60 kV on a Philips Morgagni TEM equipped with a CCD, and images were collected at magnifications of 36,000. Normality was evaluated using the Kolmogorov–Smirnov Test. Mitochondrial number was found to be normally distributed, while the distribution of mitochondrial length was non-normal. Mitochondrial number was further analyzed using a Student’s *t*-test. Mitochondrial length was further analyzed using the non-parametric Mann–Whitney test.

### 2.10. RNA-Seq Workflow and Gene Network Analysis

RNA samples from WT and Rlip^+/−^ mouse brains were prepared using the RNeasy mini kit from Qiagen (Valencia, CA, USA) according to the manufacturer’s instructions. RNA quality was assessed by microfluidic capillary electrophoresis using an Agilent 2100 Bioanalyzer (Agilent Technologies, Santa Clara, CA, USA). Ribosomal RNA was removed from total RNA using the RiboZero kit from Illumina (San Diego, CA, USA), and the resulting RNA was ethanol precipitated before cDNA synthesis. Following first-strand cDNA synthesis by reverse transcription, the second-strand synthesis was performed using DNA polymerase I and RNase H. cDNA was end repaired and 3′ end adenylated. Universal adapters were ligated, followed by 10 cycles of PCR using Illumina PCR Primer Cocktail and Phusion DNA polymerase from Illumina. Subsequent library purification with Agencourt AMPure XP beads was validated with Agilent Bioanalyzer 2100 and quantified using a Qubit from Thermo Fisher (Waltham, MA, USA). Sequencing was conducted on an Illumina HiSeq 2500 with single-end 50 bp reads. Reads were aligned using Tophat v2.0 to mouse reference genome mm9. The expression level of RefSeq genes was counted and normalized using the TMM method [42,63], and differential expression analysis was conducted using a linear model based on negative binomial distribution using “edgeR”. RPKM (reads per kilobases per million mapped reads) was defined as a number of reads/(gene length/1000 × total number of reads/1,000,000). Analyses were performed by censoring the lowest expressed genes or using log_2_(RPKM + 0.1) expression levels. 

To satisfy the criteria for differential expression, a *p* value ≤ 0.01, fold change ≥ 2, and RPKM ≥ 1 in at least 2 samples was required. Differentially expressed genes were analyzed using Ingenuity Pathway Analysis (IPA) software version 20.0 to identify canonical pathways affected by Rlip knockout and generate pathway illustrations. IPA is a commercially available bioinformatic platform from Qiagen (Hilden, Germany) which is continually updated using results curated from published literature [64].

## 3. Results

### 3.1. Rlip^+/−^ Mice Exhibit Neurocognitive Abnormalities That Resemble an AD Mouse Model

Behavioral, basic motor and sensory function tests in mouse models can reveal cognitive impairments. Since markers of OS have been shown to be significantly elevated in Rlip^+/−^ mice [44,65], we suspected that oxidative damage in the brain might lead to cognitive impairments in Rlip^+/−^ mice. Therefore, we administered behavioral tests on 5 WT and 5 Rlip^+/−^ mice, using the Open Field, Rotarod, and Morris Water Maze tests. 

**Figure 1:**
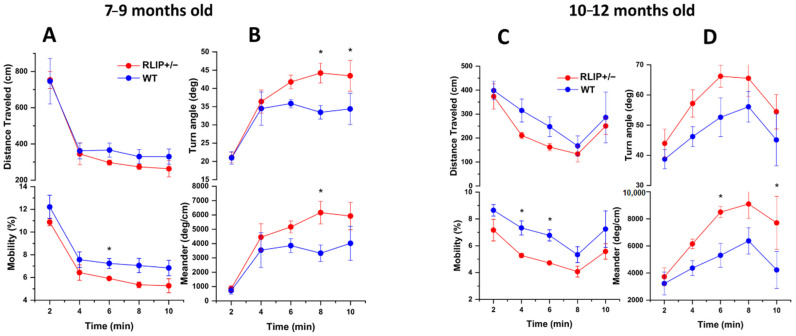
Behavioral differences between Rlip^+/−^ and wildtype (WT) mice increase with age. (**A**) In a study of 5 Rlip^+/−^ mice and 5 WT mice of 7–9 months of age, both groups exhibited habituation over the course of 10 min with reduced distance traveled (F(4,32) = 30.8; *p* < 0.0001) and mobility (F(2.2,17.9) = 37.35; *p* < 0.0001). Rlip^+/−^ mice were less mobile than WT mice (F(1,8) = 7.4, *p* = 0.0260). (**B**) Rlip^+/−^ mice tended to exhibit shallower turn angles in comparison to WT, which trended towards significance for both turn angle (F(1,8) = 5.2, *p* = 0.051), and, correcting for distance traveled, meander (F(1,8) = 4.5, *p* = 0.067). (**C**) In a study of 5 Rlip^+/−^ mice and 5 WT mice of 10–12 months of age, both groups exhibited habituation over the course of 10 min with reduced distance traveled (F(4,32) = 7.62; *p* = 0.0002) and mobility (F(4,32) = 7.38; *p* = 0.0003). (**D**) Rlip^+/−^ mice tended to exhibit a shallower turn angle compared to WT, which was more pronounced in aged mice (F(1,8) = 8.12, *p* = 0.022), and, correcting for distance traveled, increased meander (F(1,8) = 17.0, *p* = 0.0033). Significant differences (*p* < 0.05) at specific time points from post-hoc analysis (Fisher’s Least Significant Difference) are indicated with asterisks (*).

Open Field Test studies support the emergence of a potential AD or AD-like phenotype in Rlip^+/−^ mice by 7–9 months of age. Rlip^+/−^ mice were less mobile than WT mice (F(1,8) = 7.4, *p* = 0.0260) (Figure 1A). Rlip^+/−^ mice tended to exhibit shallower turn angles in comparison to WT. The differences trended towards significance for both turn angle (F(1,8) = 5.2, *p* = 0.051), and, correcting for distance traveled, meander (F(1,8) = 4.5, *p* = 0.067) (Figure 1B). These findings were extended in additional studies, showing that the differences in distance traveled (*p* = 0.0002), mobility (*p* = 0.0003), turn angle (*p* = 0.0.022) and meander (*p* = 0.0033) became more pronounced in the same mice with increased age (10–12 months), suggestive of accelerated aging in Rlip^+/−^ mice (Figure 1C,D). There was also a trend for Rlip^+/−^ mice to spend more time in the periphery than in the center of the field, relative to WT mice (*p* = 0.0607), suggestive of an emerging anxiety phenotype.

**Figure 2:**
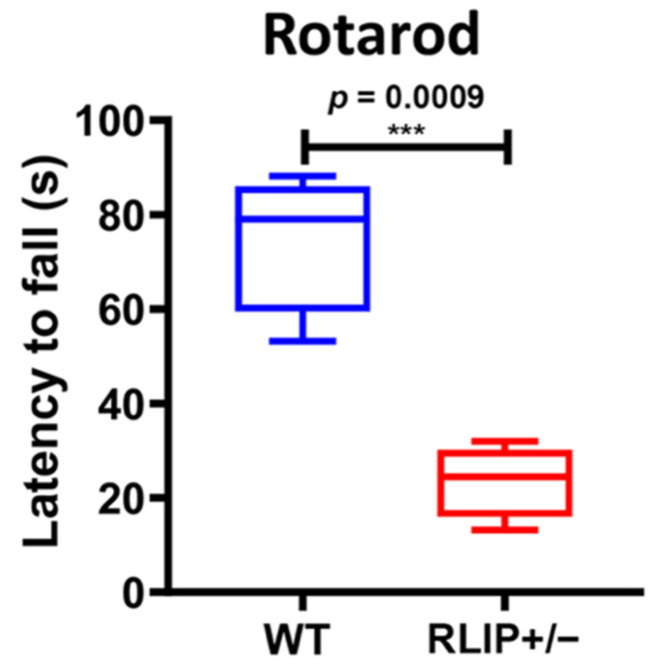
Rlip deficiency decreases the latency to fall of mice on Rotarod performance testing. Analysis of locomotor activity was measured by latency to fall off a spinning rotarod for 5 Rlip^+/−^ mice and 5 wildtype (WT) mice of 10–12 months of age. Latency to fall was significantly decreased in Rlip^+/−^ mice (*p* = 0.0009, post-hoc analysis by Fisher’s Least Significant Difference). *** indicates *p* < 0.001.

We then tested these mice in learning and memory tests. The Rotarod performance test was used to evaluate WT and Rlip^+/−^ mouse behavioral task performance, a natural fear of falling, motor coordination and fatigue. Mice were placed on a rod that rotates at 18 RPM for up to 5 min. As shown in Figure 2, Rlip haploinsufficiency negatively affected the performance of the mice. A Student’s *t*-test revealed a significant drop in the performance time on the apparatus by Rlip^+/−^ mice (mean = 23.55 s), when compared with WT cohorts (mean = 74.85 s, *p* = 0.0009).

**Figure 3:**
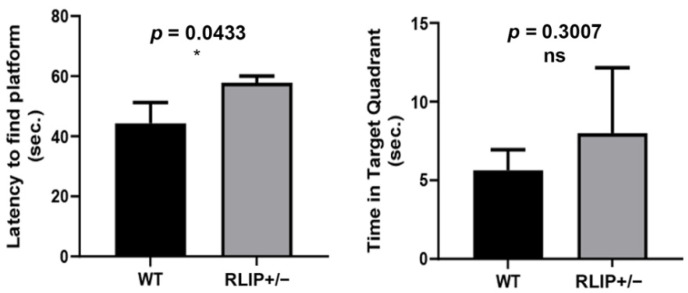
Rlip deficiency alters the willingness of mice for spatial learning. Spatial learning in 10–12-month-old mice was assessed by the Morris Water Maze test as cognitive function measure. Mean latency to find the platform and mean time spent in target quadrant are shown (*n* = 5 animals of each genotype). Significance was determined by post-hoc analysis (Fisher’s Least Significant Difference). * indicates *p* < 0.05 and ns indicates not significant.

In the Morris Water Maze test, latency to find the platform was significantly increased for Rlip^+/−^ mice (*p* = 0.0433) relative to age-matched WT mice (Figure 3). The time spent in the target quadrant was changed, but not significantly, for Rlip^+/−^ mice (*p* = 0.3007) compared to age-matched, WT mice. These observations indicate that Rlip^+/−^ mice showed impaired cognitive function (Figure 3). 

### 3.2. Effect of Rlip Deficiency on the mRNA Expression of Genes That Regulate Mitochondrial Fission/Fusion and Synaptic Function

#### 3.2.1. Mitochondrial Dynamics

**Figure 4:**
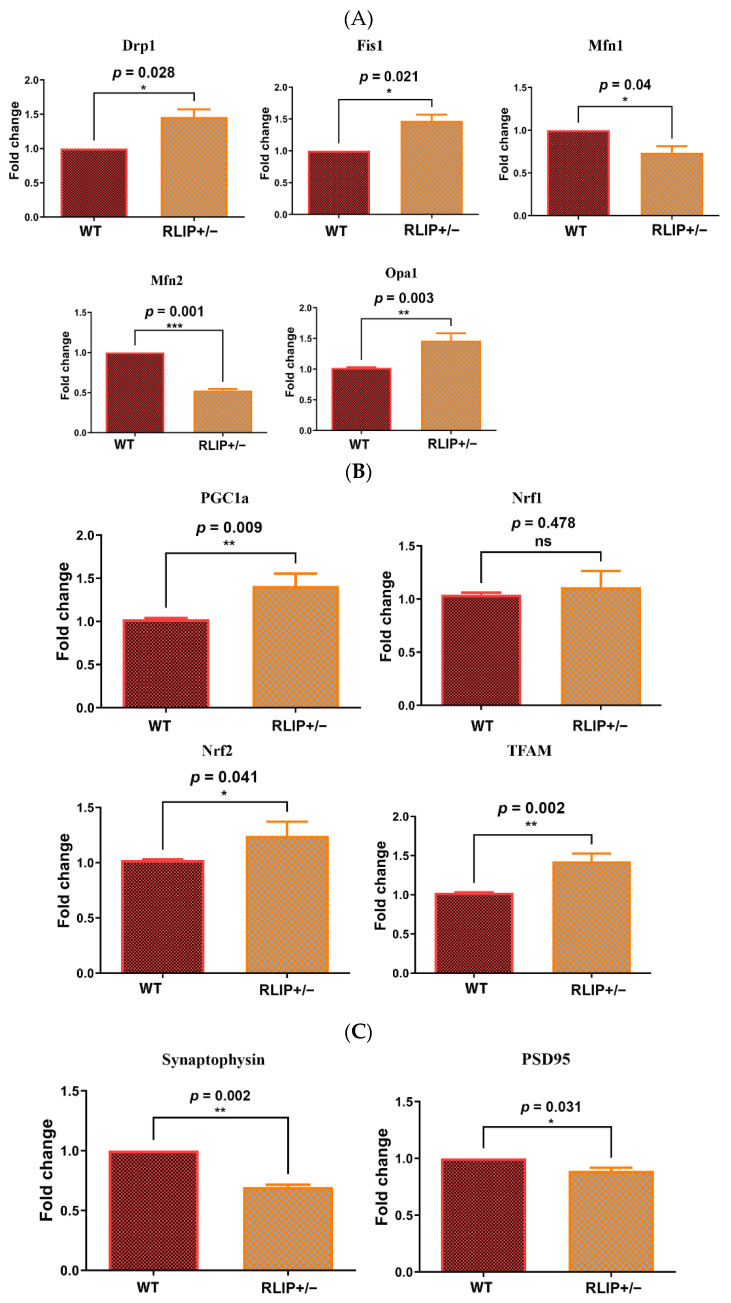
(**A**) Effect of Rlip deficiency on expression of genes that regulate mitochondrial dynamics. qRT-PCR was performed by previously described methods on mouse brain homogenates from five wildtype (WT) and five Rlip^+/−^ mice. Levels of transcripts encoding mitochondrial dynamics proteins were measured in the cerebral cortex of WT and Rlip^+/−^ animals. Gene expression levels were normalized to the beta-actin transcript and calculated as described in Materials and Methods. Asterisks denote statistically significant differences between WT and Rlip^+/−^ groups of animals, compared using a Student’s *t*-test. (**B**). Effect of Rlip deficiency on expression of genes that regulate mitochondrial biogenesis. qRT-PCR was performed by previously described methods on mouse brain homogenates from 5 WT and 5 Rlip^+/−^ mice. Levels of transcripts encoding mitochondrial biogenesis proteins were measured in the cerebral cortex of WT and Rlip^+/−^ animals. (**C**) Effect of Rlip deficiency on expression of synaptic genes. qRT-PCR was performed by previously described methods on mouse brain homogenates from 5 WT and 5 Rlip^+/−^ mice. Levels of transcripts encoding synaptic proteins were measured in the cerebral cortex of WT and Rlip^+/−^ animals. * indicates *p* < 0.05, ** indicates *p* < 0.01, *** indicates *p* < 0.001, and ns indicates not significant.

Rlip, along with RALA, has been reported to facilitate mitochondrial fission and segregation during mitosis in HeLa cells [43]. To determine whether Rlip deficiency affects mitochondrial dynamics (fission and fusion) in our model, we used quantitative real-time PCR (qRT-PCR) to measure the mRNA levels of mitochondrial fission genes (Drp1 and Fis1) and fusion genes (Mfn2, Mfn2 and Opa1) in the cerebral cortex of Rlip^+/−^ mice and age-matched control WT mice. As shown in Figure 4A, the fission genes Drp1 (*p* = 0.028) and Fis1 (*p* = 0.021) were significantly increased in Rlip^+/−^ mice relative to WT mice. On the other hand, mitochondrial fusion genes (Mfn1, *p* = 0.040; Mfn2, *p* = 0.001) were significantly reduced in Rlip^+/−^ mice relative to WT mice. However, mRNA levels of Opa1 were increased in Rlip^+/−^ mice.

Overall, these observations strongly suggest the presence of impaired mitochondrial dynamics in Rlip^+/−^ mice, similar to AD cells, APP transgenic mice [66] and Tau transgenic mice [67].

#### 3.2.2. Mitochondrial Biogenesis

mRNA expression of mitochondrial biogenesis genes was assessed in Rlip^+/−^ mice. Interestingly, mitochondrial biogenesis genes (PGC1a, *p* = 0.009; Nrf2 *p* = 0.041; and TFAM, *p* = 0.002) were increased in Rlip^+/−^ mice relative to WT mice (Figure 4B). These observations indicate that increased mitochondrial biogenesis may be a compensatory response to impaired mitochondrial function in Rlip^+/−^ mice. 

#### 3.2.3. Synaptic Genes

As shown in Figure 4C, mRNA transcripts of the synaptic genes, synaptophysin (*p* = 0.002) and PSD95 (*p* = 0.031) were significantly reduced in Rlip^+/−^ mice. 

These observations indicate that mRNA levels of mitochondrial dynamics, biogenesis, and synaptic genes in Rlip^+/−^ mice were altered similarly to other established mouse models of AD. 

### 3.3. Immunoblotting Analysis

To determine the impact of Rlip depletion on mitochondrial dynamics, mitochondrial biogenesis, and synaptic proteins, we performed immunoblotting analysis using protein lysates prepared from the cerebral cortex of WT and Rlip^+/−^ mice.

#### 3.3.1. Mitochondrial Dynamics

**Figure 5:**
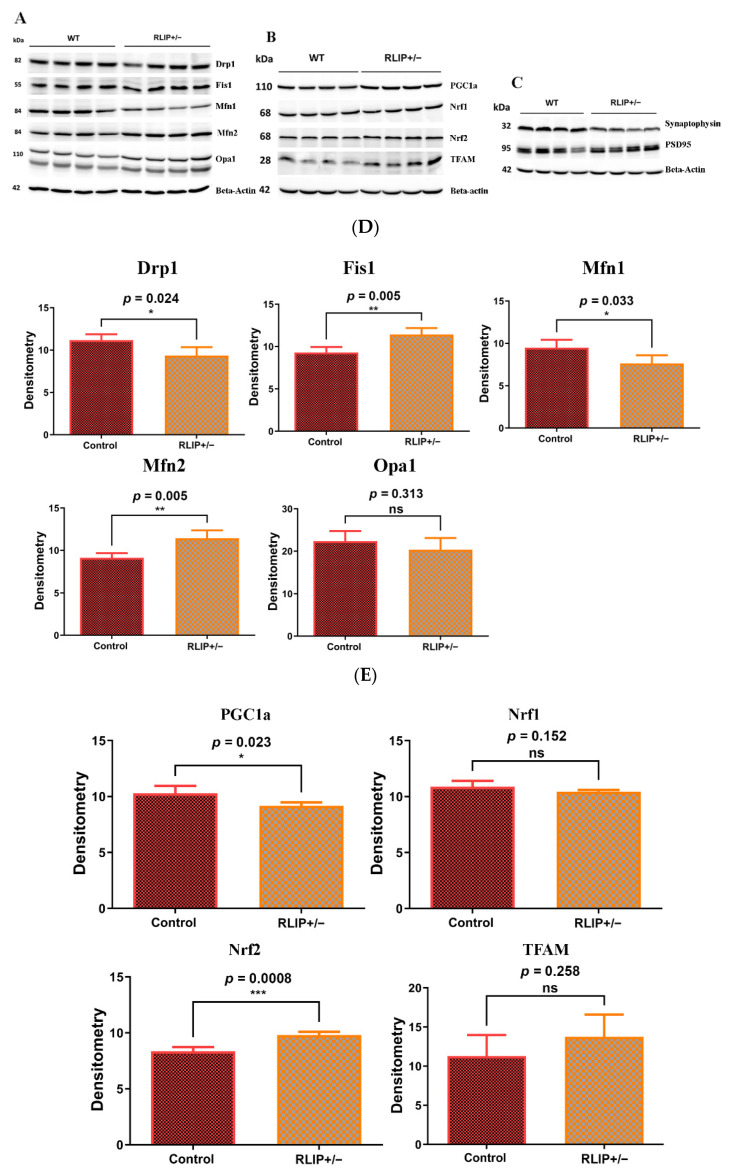

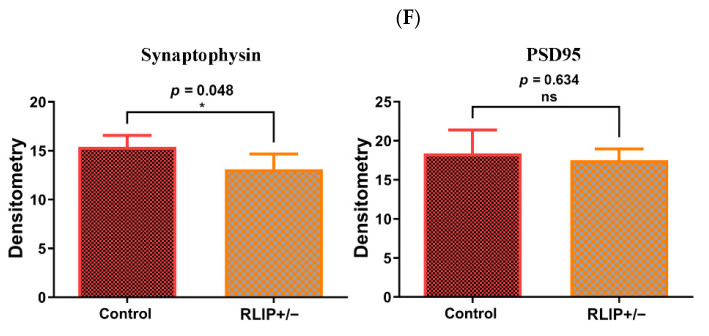
Expression of key mitochondrial proteins in the brain of wildtype (WT) and Rlip+/− mice. Rlip deficiency alters proteins regulating (A) mitochondrial dynamics, (B) mitochondrial biogenesis and (C) synaptic functions. Brain homogenates from WT and Rlip+/− mice were examined by Western blot analysis (A–C). Quantitation of the protein bands by densitometry, normalized to β-actin using ImageJ software (developed by NIH), is shown in (D–F) to indicate the average expression levels of proteins. Asterisks denote statistically significant differences between WT and Rlip+/− groups of animals, compared using Student’s *t*-test. (D) Quantitation of the mitochondrial dynamics bands by densitometry, normalized to β-actin. (E) Quantitation of the mitochondrial biogenesis bands by densitometry, normalized to β-actin. (F) Quantitation of synaptic protein bands by densitometry, normalized to β-actin. * indicates *p* < 0.05, ** indicates *p* < 0.01, *** indicates *p* < 0.001, and ns indicates not significant.

As shown in Figure 5A,D, we found a significant increase in Fis1 (*p* = 0.005) in the brains of the Rlip^+/−^ mice when compared with their WT counterparts. Mitochondrial fusion protein Mfn1 was reduced (*p* = 0.033), while Mfn2 was significantly increased (*p* = 0.005) in Rlip^+/−^ mice relative to WT mice. 

#### 3.3.2. Mitochondrial Biogenesis

A significant decrease in mitochondrial biogenesis protein PGC1a (*p* = 0.023) and a significant increase in Nrf2 expression (*p* = 0.0008) were found in Rlip^+/−^ mice relative to WT mice (Figure 5B,E). However, protein levels of Nrf1 and TFAM were not significantly different in Rlip^+/−^ mice. TFAM expression showed high variation between mice, possibly explaining the lack of statistical significance despite the apparent moderate increase in expression. Together, these observations indicate that biogenesis is affected by reduced Rlip in mice.

#### 3.3.3. Synaptic Proteins

Similar to mRNA levels, the synaptic protein synaptophysin (*p* = 0.048) was significantly reduced in Rlip^+/−^ mice when compared with WT mice (Figure 5C,F). PSD95 protein expression appeared to be slightly reduced in Rlip^+/−^ mice, but this change was not significant (*p* = 0.634). These results indicate that synaptic activity is likely defective in Rlip^+/−^ mice, a result that has also been observed in APP and tau transgenic mouse models of AD [66,67]. 

#### 3.3.4. Rlip in Post Mortem Brains

Rlip protein was measured by immunoblot in the prefrontal cortex of five early-stage AD brains and four normal brains. Rlip protein expression was not elevated in the prefrontal cortex of the early-stage AD brains relative to the cortex of normal brains (Supplementary Figure S1). This result suggests that the disruption of Rlip by expression level changes is not commonly present in early stage AD.

### 3.4. Immunofluorescence Analysis

#### 3.4.1. Mitochondrial Dynamics Proteins

**Figure 6:**
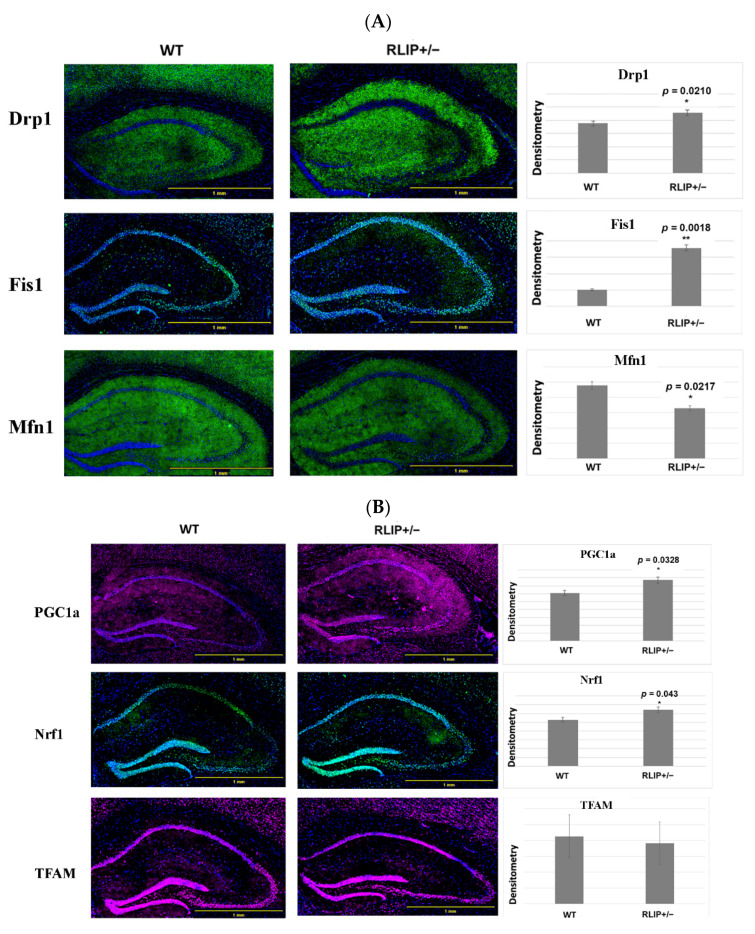

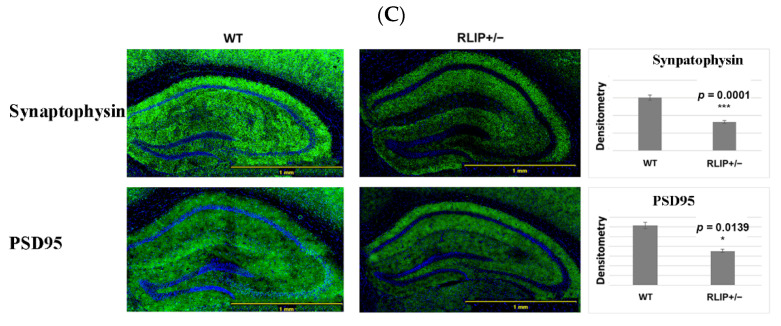
(**A**) Immunofluorescence analysis of mitochondrial dynamics in the neurons of the hippocampi of wildtype (WT) and Rlip^+/−^ mice. A comparison of hippocampal expression of mitochondrial dynamics and proteins between WT and Rlip^+/−^ mice showed significant increases in Drp1, Fis1 and a decrease in Mfn1 expression in Rlip^+/−^ mice compared with WT mice. (**B**) Immunofluorescence analysis of mitochondrial biogenesis proteins in the neurons of the hippocampi of WT and Rlip^+/−^ mice. A comparison of hippocampal expression of mitochondrial biogenesis proteins between WT and Rlip^+/−^ mice showed significant increases in PGC1a and Nrf1 expression in Rlip^+/−^ mice compared with WT mice. (**C**) Immunofluorescence analysis of synaptic proteins in the neurons of the hippocampi of WT and Rlip^+/−^ mice. A comparison of hippocampal expression of synaptic proteins between WT and Rlip^+/−^ mice showed significant decreases in synaptophysin and PSD95 expression in Rlip^+/−^ mice compared with WT mice. * indicates *p* < 0.05, ** indicates *p* < 0.01, and *** indicates *p* < 0.001.

We performed immunofluorescence microscopy on mitochondrial dynamics, mitochondrial biogenesis, and synaptic proteins to understand the impact on the hippocampus in Rlip^+/−^ mice. As shown in Figure 6A, the immunoreactivities of Drp1 (*p* = 0.0210) and Fis1 (*p* = 0.0018) were significantly increased in the Rlip^+/−^ mice relative to WT mice. On the other hand, the mitochondrial fusion protein Mfn1 was significantly reduced in Rlip^+/−^ mice (*p* = 0.0217) relative to WT mice. Aside from Drp1, which showed increases by qRT-PCR and immunofluorescence but a decrease by Western blot, our immunofluorescence data concur with qRT-PCR (mRNA levels) and immunoblotting (protein levels) data from the cerebral cortex. In the case of Drp1, it is possible that the expression changes are region-specific. Immunofluorescence signal was quantified for the hippocampus, while the Western blots and qRT-PCR were performed using protein lysates from the cerebral cotex.

#### 3.4.2. Mitochondrial Biogenesis

Figure 6B indicates significantly increased immunoreactivities of the mitochondrial biogenesis proteins PGC1a (*p* = 0.0328) and Nrf1 (*p* = 0.043) in Rlip^+/−^ mice compared to WT mice. However, the immunoreactivity of TFAM did not change in Rlip^+/−^ mice when compared to WT mice. As with Drp1, Nrf1 and PGC1a were heterogeneously affected in Rlip^+/−^ mice across the qRT-PCR, immunoblot and immunofluorescence results, suggesting region-specific variation or post-transcriptional regulatory events, respectively. 

#### 3.4.3. Synaptic Proteins

As shown in Figure 6C, synaptic proteins, including synaptophysin (*p* = 0.0001) and PSD95 (*p* = 0.0139), were significantly reduced in Rlip^+/−^ mice, a result that is similar to the mRNA and immunoblot results and similar to previously published immunofluorescence data on the APP and tau transgenic mouse models of AD [66,67]. It is worth noting that synaptophysin immunoreactivity was reduced highly significantly. 

Overall, our qRT-PCR, immunoblot, and immunofluorescence results indicate that mitochondrial fission is increased and mitochondrial fusion and synaptic function are impaired in Rlip^+/−^ mice.

### 3.5. Rlip Haploinsufficiency Reduces the Activity of OS and Nrf2-Related Enzymes in the Mouse Brain

In our previous studies, we found that Rlip depletion induced OS in cells and in several mouse tissues [68,69,70,71]. Although the brain represents only 2% of body weight, it consumes 20% of the total body oxygen [72]. Therefore, the brain should be highly susceptible to oxidative stress following treatments which impair the detoxification of reactive oxygen species. We previously analyzed OS markers in wildtype and Rlip-deficient mouse brain tissue. We demonstrated that LPO and total reactive aldehyde (TBARS, thiobarbituric acid reactive substances) were higher in the Rlip^+/−^ and Rlip^−/−^ mice than in WT mice [44,65,71]. These data suggest the presence of elevated OS in the brains of Rlip-depleted mice. Therefore, we investigated the effects of Rlip knockout on Nrf2-related antioxidant defenses.

**Figure 7:**
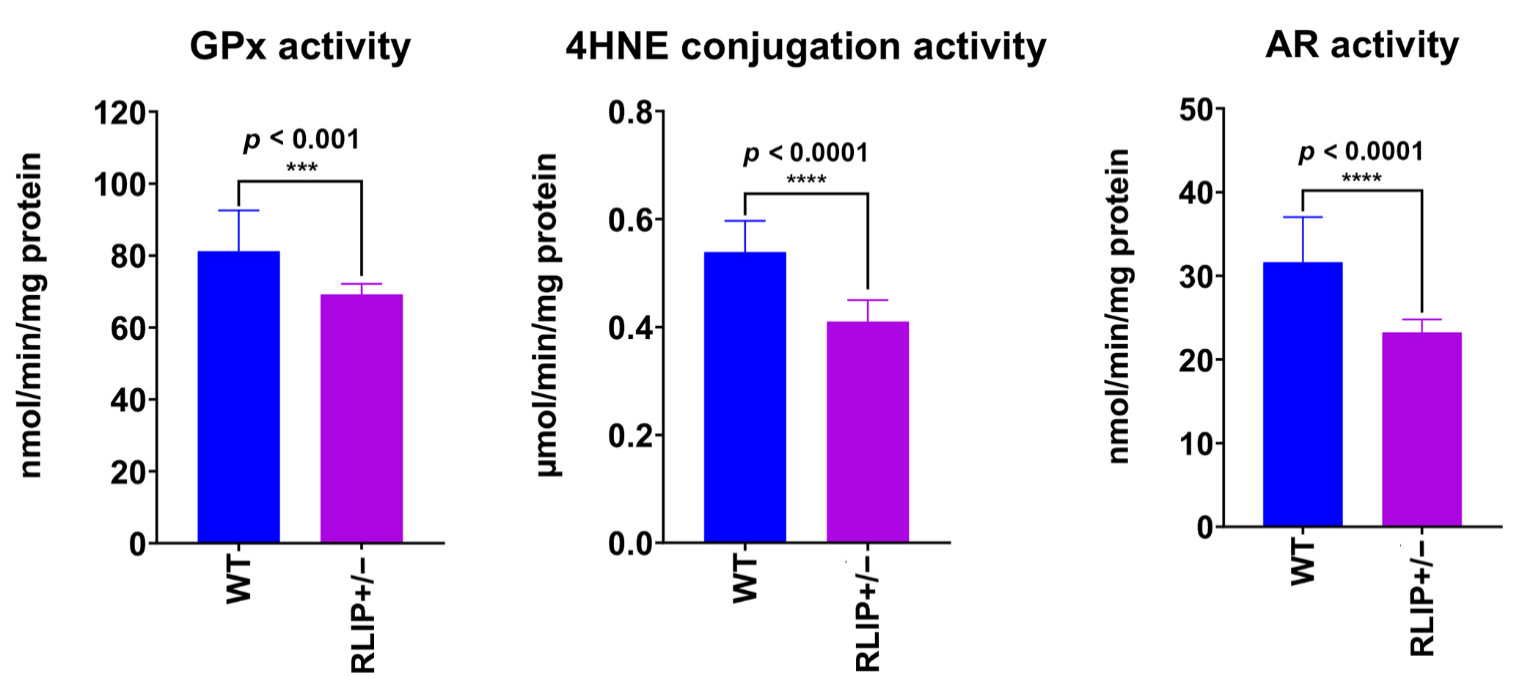
Antioxidant enzyme activities in Rlip^+/−^ mouse brains are lower than in corresponding control wildtype mice. Brain tissue homogenates were prepared from three wildtype mice and three Rlip^+/−^ mice, and enzyme activity was measured in triplicate in individual homogenates using spectrophotometric assays as described in Methods. The difference in enzyme activity between WT and Rlip^+/−^ mice was significant for all tissues studied. *** indicates *p* < 0.001 and **** indicates *p* < 0.0001.

Glutathione peroxidase (GPx) is a mitochondrial antioxidant enzyme which scavenges free radicals and generally helps to maintain mitochondrial function. We measured GPx enzymatic activity in Rlip^+/−^ and WT mice. As expected, GPx activity was significantly reduced in Rlip^+/−^ mice relative to WT (*p* < 0.001), supporting a loss of mitochondrial function in Rlip^+/−^ mice (Figure 7). 

Significantly reduced rates of GSH conjugation to 4HNE were also observed in Rlip^+/−^ mice when compared with WT mice, indicating reduced activity of glutathione transferase GSTA4, the enzyme responsible for conjugation of 4-HNE with glutathione (*p* < 0.0001). Finally, aldose reductase (AR) activity was significantly reduced in Rlip^+/−^ mice compared to WT mice (*p* < 0.0001).

Our results confirmed lower activities of the Nrf2-regulated antioxidant enzymes in Rlip^+/−^ mice compared with age-matched WT mice (Figure 7). Consistently, reduced activity of GST, GR, GPx, GGCS, GGT and G6PD in the brain and other tissues of Rlip knockout mice has been reported previously [68,69,70,71]. 

### 3.6. Electron Micrographs Demonstrate Aberrant Mitochondria in Rlip^+/−^ Mice

**Figure 8:**
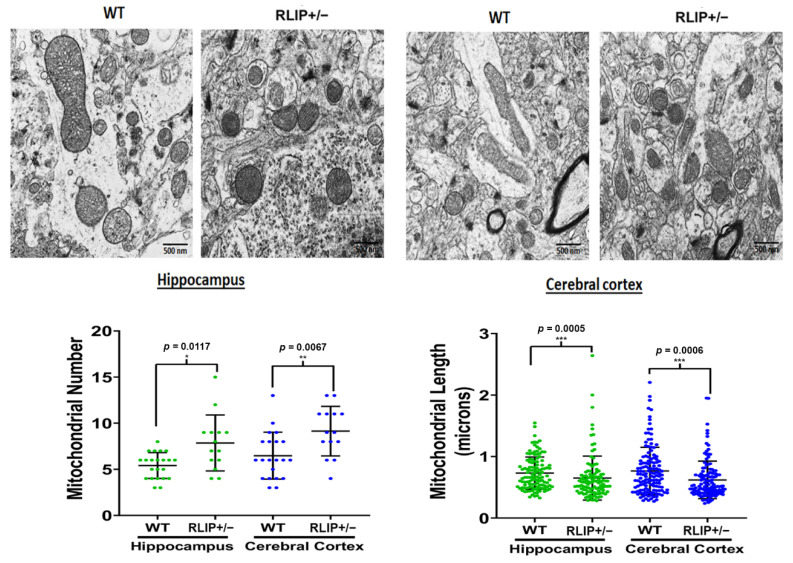
Electron micrographs demonstrating changes in mitochondria number and length in Rlip^+/−^ mice relative to wildtype (WT) mice. Transmission electron microscopy was performed using hippocampal and cortical tissues from 10-month-old Rlip^+/−^ (*n* = 5) and wildtype mice (*n* = 5). Mitochondrial number and length were assessed using previously published methods [67,74]. * indicates *p* < 0.05, ** indicates *p* < 0.01, and *** indicates *p* < 0.001.

Mitochondria are the primary source of energy for neurons. Additionally, OS-associated mitochondrial dysfunction can be caused by changes in mitochondrial ultrastructure due to oxidative damage. To determine whether Rlip haploinsufficiency induces changes in mitochondrial ultrastructure, we compared mitochondrial morphology (number and length) in hippocampi and cerebral cortices from WT and Rlip^+/−^ mice using transmission electron microscopy. Electron microscopy revealed that mitochondrial number was significantly increased in the hippocampi (*p* = 0.0117) and cortices (*p* = 0.0067) from Rlip^+/−^ mice when compared with WT mice (Figure 8). The mitochondrial length was significantly reduced in both the hippocampi (*p* = 0.0005) and the cerebral cortices (*p* = 0.0006). Overall, the differences in mitochondrial size, morphology and number were quite striking, similar to published reports in AD cell [73,74] and mouse models [66,67]. These results are consistent with our results showing increased expression of mitochondrial fission proteins and decreased expression of mitofusins (Figure 4, Figure 5 and Figure 6). Taken together, these results suggest that Rlip^+/−^ mice also have OS-associated mitochondrial dysfunction, adding further validation to this knockout model. 

### 3.7. Transcriptional Effect of Rlip Deficiency on Mitochondrial Function Pathways and CREB Signaling Pathways

**Figure 9:**
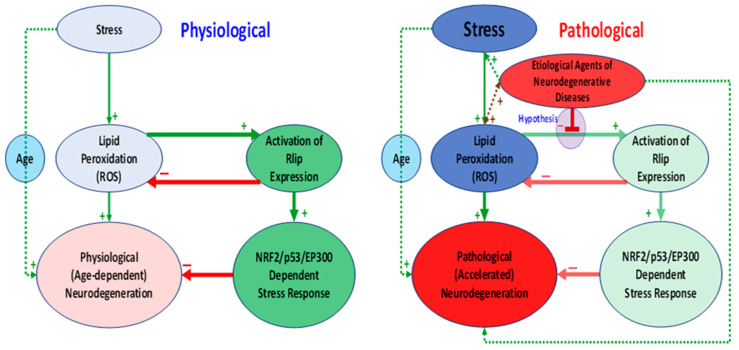
A hypothetical model of the role of Rlip-regulated expression of stress-defense genes controlled by Nrf2, p53 and EP300. Red arrows represent inhibition, and green arrows represent activation. Note that according to this hypothesis, the etiological agents of neurodegenerative diseases inhibit activation of Rlip expression, which normally inhibits lipid peroxidation and neurodegeneration. Red shaded ovals indicated pathological effects and green shaded ovals indicate effects which promote neuronal health. More saturated shades indicated a greater effect strength and lighter shades indicate a reduced effect strength.

Preliminary evidence for the role of Rlip in neural synapses remains indirect. Though we did observe a reduction of synaptophysin expression, we have not yet visualized the neuronal synapses, studied clathrin-dependent endocytosis (CDE) in them or determined synaptic functions. Given the established and accepted function of Rlip in CDE, it is likely that the proposed studies will experimentally confirm synaptic dysfunction. Analysis of RNA-seq transcriptomic results show that alterations in the canonical pathways of mitochondrial dysfunction (Figure S2) and neuronal CREB signaling (Figure S3) were highly represented among the differentially expressed genes. Mitochondrial dysfunction is associated with elevated oxidative stress, and CREB signaling is important for neuronal plasticity and oxidative stress response, all of which are implicated in AD [75,76,77]. This supports an important regulatory role of Rlip in neural functioning and the hypothesis that decreased expression of Rlip may disrupt memory formation, exacerbate neuronal damage, and lead to cognitive dysfunction. A model for how loss of Rlip could exacerbate oxidative stress and neurodegeneration is illustrated in Figure 9. 

## 4. Discussion

The long-term goal of our study is to understand the impact of reduced expression of the Rlip gene in oxidative stress and mitochondrial dysfunction in Alzheimer’s disease progression and pathogenesis. In the current study, for the first time, we studied Rlip heterozygous knockout mouse (Rlip^+/−^) brain tissues for mRNA and protein expression levels of mitochondrial dynamics, mitochondrial biogenesis and synaptic genes. We additionally measured antioxidant enzymatic activities, mitochondrial morphology, and behavioral changes in Rlip^+/−^ mice relative to WT mice. 

Our comprehensive and detailed analysis revealed that mitochondrial fission is increased, while mitochondrial fusion and the expression of synaptic proteins were reduced in Rlip^+/−^ mice. Rlip^+/−^ mice were cognitively deficient based on the Open Field, Rotarod, and Morris Water Maze tests. The activities of antioxidant enzymes, including glutathione peroxidase, aldose reductase, and glutathione s-transferase A4, were significantly reduced in Rlip^+/−^ mice relative to age-matched wildtype mice. These features are similar to the APP and Tau transgenic mouse models of Alzheimer’s disease. In this work, we studied 7–12-month-old Rlip^+/−^ mice in comparison to wildtype mice of the same genetic background. A limitation of our study is the narrow age range of the mice. It will be important in future studies to evaluate Rlip^+/−^ mice at a wide range of ages, including 2, 6, 12, 18 and 24 months, or until the terminal stages of life. 

### 4.1. Cognitive Behavior

The most predominant and striking symptom of AD in patients is the progressive decline in cognition primarily due to the loss of neurons and synapses in the brain. In the current study, we used the Morris Water Maze (MWM), Open Field and Rotarod tasks to evaluate cognitive changes in the Rlip^+/−^ mouse model. The Open Field Test is used for the measurements of general locomotor activity, exploratory behavior and measures of anxiety. Rlip^+/−^ mice showed reduced mobility, traveled less distance, and their turn angle was significantly higher. Thus, we observed significant differences between Rlip^+/−^ and wildtype mice in overall activity levels and measures of anxiety. Rlip^+/−^ mice showed a significantly higher number of freezing episodes and increased freezing time. Our behavioral data suggest that Rlip^+/−^ mice displayed increased stress or anxiety. 

The Rotarod Test suggests that Rlip^+/−^ mice had reduced motor coordination or fatigue resistance. Rlip^+/−^ mice displayed a phenotype of an AD mouse model with impairments in motor coordination and balance present at the age of 10 months, most likely due to impaired cerebellar activity and increased anxiety. Since Rlip^+/−^ mice show increases in oxidative damage and mitochondrial dysfunction and decreases in Nrf2-dependent enzyme levels, studying the behavior of these mice may contribute to the understanding of the pathophysiology of neuropsychiatric disorders. 

### 4.2. Mitochondrial and Synaptic Gene Expression and Protein Levels

Transcript levels of mitochondrial and synaptic genes in mouse brains were examined. qRT-PCR results from cerebral cortex homogenates suggest increases in Drp1 and Fis1 and decreases in Mfn1, Mfn2, synaptophysin and PSD95 mRNAs, similar to mutant HT22 cells, as previously shown by Reddy et al. [45], and the APP and Tau mouse models, as shown by the Reddy lab [66,67] and others [78,79]. Through fusion and fission events, mitochondria maintain dynamics and bioenergetics to fine-tune nutrients and electrochemical signals. The mitofusins Mfn1 and Mfn2 regulate mitochondrial fusion. Mfn1 plays a more prominent role in mitochondrial fusion, whereas Mfn2 mainly affects mitochondrial metabolism. On the other hand, mitochondrial fission is mediated by Drp1, which interacts with another adaptor, Fis1, and is known to be increased in AD patients. Overexpression of Fis1 protein in the cerebral cortex neurons of Rlip^+/−^ mice confirms the findings of our qRT-PCR analysis. Reduced expression of synaptic genes in Rlip^+/−^ mouse brains is possibly responsible for synaptic damage, and thus cognitive dysfunction, in Rlip deficient mice. Taken together, the results of mRNA and protein analyses suggest that Rlip deficiency causes dysregulation of mitochondrial dynamics and impaired synaptic health. These results suggest that dysfunctional mitochondria and loss of synaptic plasticity lead to cognitive impairment in these mice.

### 4.3. Oxidative Stress and Antioxidant Enzymes

Free radical-scavenging antioxidants and glutathione (GSH)-mediated metabolism of reactive oxygen species are defenses against neurodegeneration that are impaired in Alzheimer’s disease. Understanding their regulatory mechanisms will be of critical importance in devising novel therapies.

In the current study, we report for the first time that Rlip depletion-associated dysregulation of Nrf2 causes decreased levels of antioxidant/anti-electrophile enzymes, cognitive impairment, and abnormalities of mitochondrial structure and synaptic proteins. RNA sequencing and pathway analysis studies show that Rlip depletion epigenetically regulates several AD-linked pathways, including CREB signaling and mitochondrial function. 

The postulated role of Rlip depletion in enhancing the effects of oxidative stress in the brain raises the question of which species might lead to damaging effects. Rlip knockout decreased the metabolism of lipid hydroperoxides by glutathione peroxidase, similar to what is seen in the transgenic APP and tau mouse models of AD [66,67]. While the reduced glutathione peroxidase activity may be physiologically significant, most striking is the reduced 4-HNE conjugating activity observed in Rlip^+/−^ mouse brains. In mammals, 4-HNE is the product of non-enzymatic degradation of oxidized polyunsaturated fatty acids, primarily of the abundant arachidonic acid. Moreover, 4-hydroxyalkenals of chain lengths different from the nine carbon atoms of 4-HNE may arise from peroxidation of other polyunsaturated fatty acids. As lipid peroxidation and the subsequent decomposition of the peroxides are thought to be non-enzymatic processes driven by oxidative conditions, 4-HNE and/or similar 4-hydroxyalkenals are expected to be generated at greater levels by the oxidative stresses produced in AD brains. Lower activity of aldose reductase, a key enzyme in the polyol pathway, was found in Rlip^+/−^ mouse brains. AR catalyzes nicotinamide adenosine dinucleotide phosphate-dependent reduction of glucose to sorbitol, leading to excessive accumulation of intracellular reactive oxygen species in AD brains. Thus, reduced detoxification of 4-HNE and other oxidants may be a major physiological result of Rlip depletion that leads to dementia in these mice.

### 4.4. Mitochondrial Morphology

Mitochondrial dysfunction is an important part of AD. These energy powerhouses are capable of self-replication but become partially dysfunctional in brains with AD through a variety of mechanisms, including a continuous vicious cycle of oxidative/electrophilic stress. To further establish the role of Rlip in mitochondrial function and morphology, we assessed mitochondrial number and length in the hippocampus and cortical tissues from WT and Rlip^+/−^ mice. Results suggest that mitochondrial number and length are quite strikingly different in Rlip^+/−^ mice. Specifically, Rlip^+/−^ mice appear to have an increased number of mitochondria of smaller sizes relative to WT mice. This result is consistent with our qRT-PCR, immunoblot, and immunofluorescence microscopy data showing that mitochondrial fission proteins generally have increased expression in Rlip^+/−^ mice, while the expression of mitofusins was generally decreased. 

### 4.5. RNA-Seq Data

Our RNA-seq data demonstrate overlap in the gene networks of AD and aging in Rlip-deficient mice. Because most of these genes are stress-induced, elevated oxidative stress in Rlip knockout mice should have caused upregulation; the experimental observations showed the opposite. Thus, we offer a provocative explanation: Rlip deficiency impairs the normal stress-induced transcriptional activation of many genes associated with AD by disrupting the ability of Nrf2 to respond to oxidative stress. This is based on indirect evidence, and merits further examination. APP mutant mice also have defective Nrf2 regulation which is corrected by mitochondria-targeted molecules SS31, MitoQ, DDQ, and others, such as citalopram and curcumin, which reduce AD pathology in cell and mouse models [50,73,80,81,82].

In summary, our initial characterization of Rlip^+/−^ mice revealed several aspects of oxidative stress, mitochondrial, and synaptic deficits similar to transgenic APP and Tau mouse models of AD. Our initial observations are exciting and warrant further analysis of the time course of cognitive decline in Rlip^+/−^ mice. Additionally, we are currently crossing Rlip knockout mice with several APP and Tau transgenic mouse strains to evaluate whether reduced Rlip expression exacerbates cognitive decline in these strains. Alternatively, if a partial deficiency of Rlip causes behavioral abnormalities, oxidative stress, mitochondrial dysfunction and synaptic dysfunction, it will also be worthwhile in future studies to evaluate the cognitive behavior, oxidative stress, and mitochondrial and synaptic activities in transgenic mice engineered to overexpress Rlip. Importantly, future efforts to examine whether Rlip overexpression can reduce the cognitive dysfunction, mitochondrial aberrations, and synaptic deficits developed by transgenic APP and Tau mice may lead to novel therapeutic options for AD patients. Finally, we have begun examining the brains of AD patients and normal controls for evidence of Rlip-related differences. In Western blots of prefrontal cortices from early-stage AD patients, we did not find a significant change in the expression level of Rlip. In future studies, we will examine post-mortem cortex and hippocampal tissues in a larger number of late-stage AD patients to determine whether changes in Rlip expression or localization develop later in disease progression or whether abnormalities in Rlip only occur in certain subsets of patients. These and other studies will be crucial in determining whether Rlip expression or regulation plays a role in the natural etiology of AD, whether Rlip-based interventions could be protective against the progression of AD, or whether the Rlip^+/−^ mouse model should simply be used as a tool with which to study the impacts of elevated oxidative stress on the progression of AD.

## Figures and Tables

**Table 1:** Summary of qRT-PCR oligonucleotide primers used in measuring mRNA expression of mitochondrial dynamics, mitochondrial biogenesis, and synaptic genes.

Gene	DNA Sequence (5′–3′)
	Mitochondrial dynamics genes
Drp1	Forward primer ATGCCAGCAAGTCCACAGAA
	Reverse primer TGTTCTCGGGCAGACAGTTT
Fis1	Forward primer CAAAGAGGAACAGCGGGACT
	Reverse primer ACAGCCCTCGCACATACTTT
Mfn1	Forward primer GCAGACAGCACATGGAGAGA
	Reverse primer GATCCGATTCCGAGCTTCCG
Mfn2	Forward primer TGCACCGCCATATAGAGGAAG
	Reverse primer TCTGCAGTGAACTGGCAATG
Opa1	Forward primer ACCTTGCCAGTTTAGCTCCC
	Reverse primer TTGGGACCTGCAGTGAAGAA
	Mitochondrial biogenesis genes
PGC1a	Forward primer GCAGTCGCAACATGCTCAAG
	Reverse primer GGGAACCCTTGGGGTCATTT
Nrf1	Forward primer AGAAACGGAAACGGCCTCAT
	Reverse primer CATCCAACGTGGCTCTGAGT
Nrf2	Forward primer ATGGAGCAAGTTTGGCAGGA
	Reverse primer GCTGGGAACAGCGGTAGTAT
TFAM	Forward primer TCCACAGAACAGCTACCCAA
	Reverse primer CCACAGGGCTGCAATTTTCC
	Reverse primer AGACGGTTGTTGATTAGGCGT
	Synaptic genes
Synaptophysin	Forward primer CTGCGTTAAAGGGGGCACTA
	Reverse primer ACAGCCACGGTGACAAAGAA
PSD95	Forward primer CTTCATCCTTGCTGGGGGTC
	Reverse primer TTGCGGAGGTCAACACCATT
	Housekeeping genes
B-actin	Forward primer AGAAGCTGTGCTATGTTGCTCTA
	Reverse primer TCAGGCAGCTCATAGCTCTTC

**Table 2:** Summary of antibody dilutions used in the immunoblotting analysis of mitochondrial dynamics, mitochondrial biogenesis, and synaptic proteins.

Marker	Primary Antibody Species and Dilution	Purchased from Company (City and Country)	Secondary Antibody, Species, and Dilution
DRP1	Rabbit Polyclonal 1:1000	Proteintech Group (Rosemont, IL, USA)	Donkey anti-rabbit HRP 1:10,000
FIS1	Rabbit Polyclonal 1:500	Novus Biologicals (Centennial, CO, USA)	Donkey anti-rabbit HRP 1:10,000
MFN1	Mouse Monoclonal 1:1000	Abcam (Cambridge, UK)	Donkey anti-mouse HRP 1:10,000
MFN2	Mouse Monoclonal 1:1000	Abcam (Cambridge, UK)	Donkey anti-mouse HRP 1:10,000
OPA1	Rabbit Polyclonal 1:1000	Novus Biologicals (Centennial, CO, USA)	Donkey anti-rabbit HRP 1:10,000
PGC1α	Rabbit Polyclonal 1:1000	Novus Biologicals (Centennial, CO, USA)	Donkey anti-rabbit HRP 1:10,000
NRF1	Rabbit Polyclonal 1:1000	Cell Signaling Technology (Danvers, MA, USA)	Donkey anti-rabbit HRP 1:10,000
NRF2	Rabbit Polyclonal 1:1000	Novus Biologicals (Centennial, CO, USA)	Donkey anti-rabbit HRP 1:10,000
TFAM	Rabbit Polyclonal 1:2000	Abcam (Cambridge, UK)	Donkey anti-rabbit HRP 1:10,000
PSD95	Rabbit Polyclonal 1:1000	Cell Signaling Technology (Danvers, MA, USA)	Donkey anti-rabbit HRP 1:10,000
SYNAPTOPHYSIN	Rabbit Polyclonal 1:3000	Novus Biologicals (Centennial, CO, USA)	Donkey anti-rabbit HRP 1:10,000
RALBP1	Mouse Monoclonal 1:1000	Millipore Sigma (Burlington, MA, USA)	Goat anti-mouse HRP 1:1000
B-Actin	Mouse Monoclonal 1:2000	Millipore Sigma (Burlington, MA, USA)	Sheep anti-mouse HRP 1:10,000

**Table 3:** Summary of antibody dilutions used in the immunofluorescence analysis of mitochondrial dynamics, mitochondrial biogenesis, and synaptic proteins.

Marker	Primary Antibody Species and Dilution	Purchased from Company, City and State	Secondary Antibody, Dilution
DRP1	Rabbit Polyclonal 1:100	Novus Biologicals (Centennial, CO, USA)	Donkey anti-rabbit Alexa Fluor 488 1:200
Fis1	Rabbit Polyclonal 1:100	Novus Biologicals (Centennial, CO, USA)	Donkey anti-rabbit Alexa Fluor 488 HRP 1:200
Mfn1	Rabbit Polyclonal 1:100	Thermo Fisher Scientific (Waltham, MA, USA)	Donkey anti-rabbit Alexa Fluor 488 HRP 1:200
PGC1α	Rabbit Polyclonal 1:100	Novus Biologicals (Centennial, CO, USA)	Donkey anti-rabbit Alexa Fluor 647 HRP 1:200
NRF1	Rabbit Polyclonal 1:100	Abcam (Cambridge, UK)	Donkey anti-rabbit Alexa Fluor 488 HRP 1:200
TFAM	Rabbit Polyclonal 1:100	Novus Biologicals (Centennial, CO, USA)	Donkey anti-rabbit Alexa Fluor 647 HRP 1:200
PSD95	Rabbit Polyclonal 1:300	Cell Signaling Technology (Danvers, MA, USA)	Donkey anti-rabbit Alexa Fluor 488 HRP 1:500
Synaptophysin	Rabbit Polyclonal 1:400	Novus Biologicals (Centennial, CO, USA)	Donkey anti-rabbit Alexa Fluor 488 HRP 1:600

## Data Availability

The data presented in this study are available on request from the corresponding author.

## Supplementary Materials

The following are available online at https://www.mdpi.com/article/10.3390/cells10113113/s1, Figure S1: Rlip protein expression is not elevated in the prefrontal cortex of early-stage AD patients; Figure S2: Transcriptional effect of Rlip deficiency on mitochondria; Figure S3: Transcriptomic effect of Rlip deficiency.
