# Supplementary material for: RALBP1 in Oxidative Stress and Mitochondrial Dysfunction in Alzheimer’s Disease
Source: Cells. 2021 Nov 10;10(11):3113. doi: 10.3390/cells10113113 (PMC8620796; doi:10.3390/cells10113113)
Supplement: Supplementary file 1 [file cells-10-03113-s001.zip › cells-1457030-supplementary.pdf]

cells

Supplementary Materials

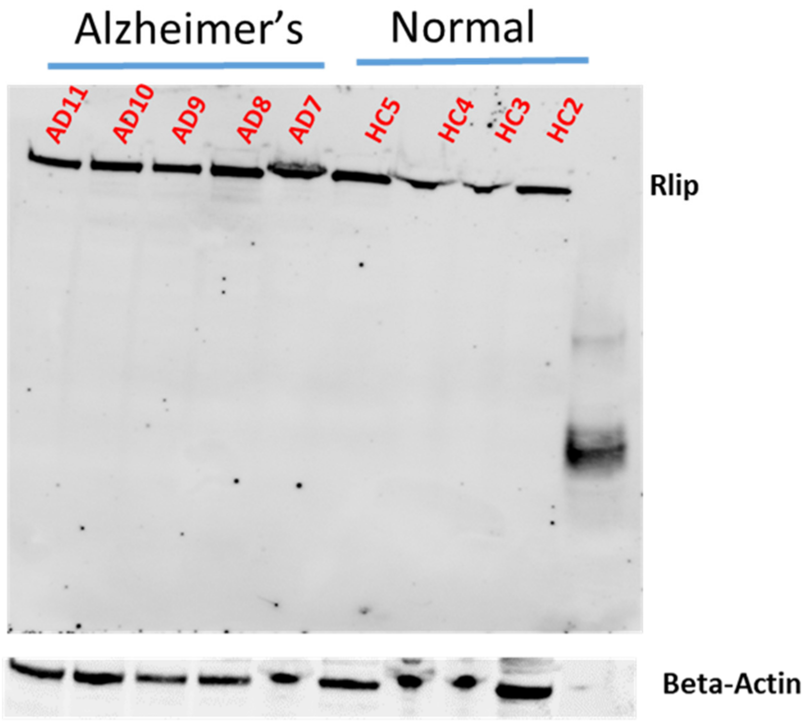

Rlip/beta actin		Mean	SD	
AD11	0.98079			
AD10	0.93888			
AD9	1.4115			
AD8	1.5512			
AD7	2.22977	1.42243	0.52378	
HC5	1.25617			
HC4	1.0861		*p* value	0.21498
HC3	1.08578			
HC2	0.7122	1.03506	0.22972	

Figure S1. Rlip protein expression is not elevated in the prefrontal cortex of early-stage Alzheimer's disease (AD) patients. Rlip protein was measured in prefrontal cortex from 5 early-stage AD and 4 normal brains. Rlip signal was normalized to that of beta-actin. Densitometry for each lane is shown in the table. There was no significant difference in the expression level by Student's *t*-test. SD: Standard Deviation

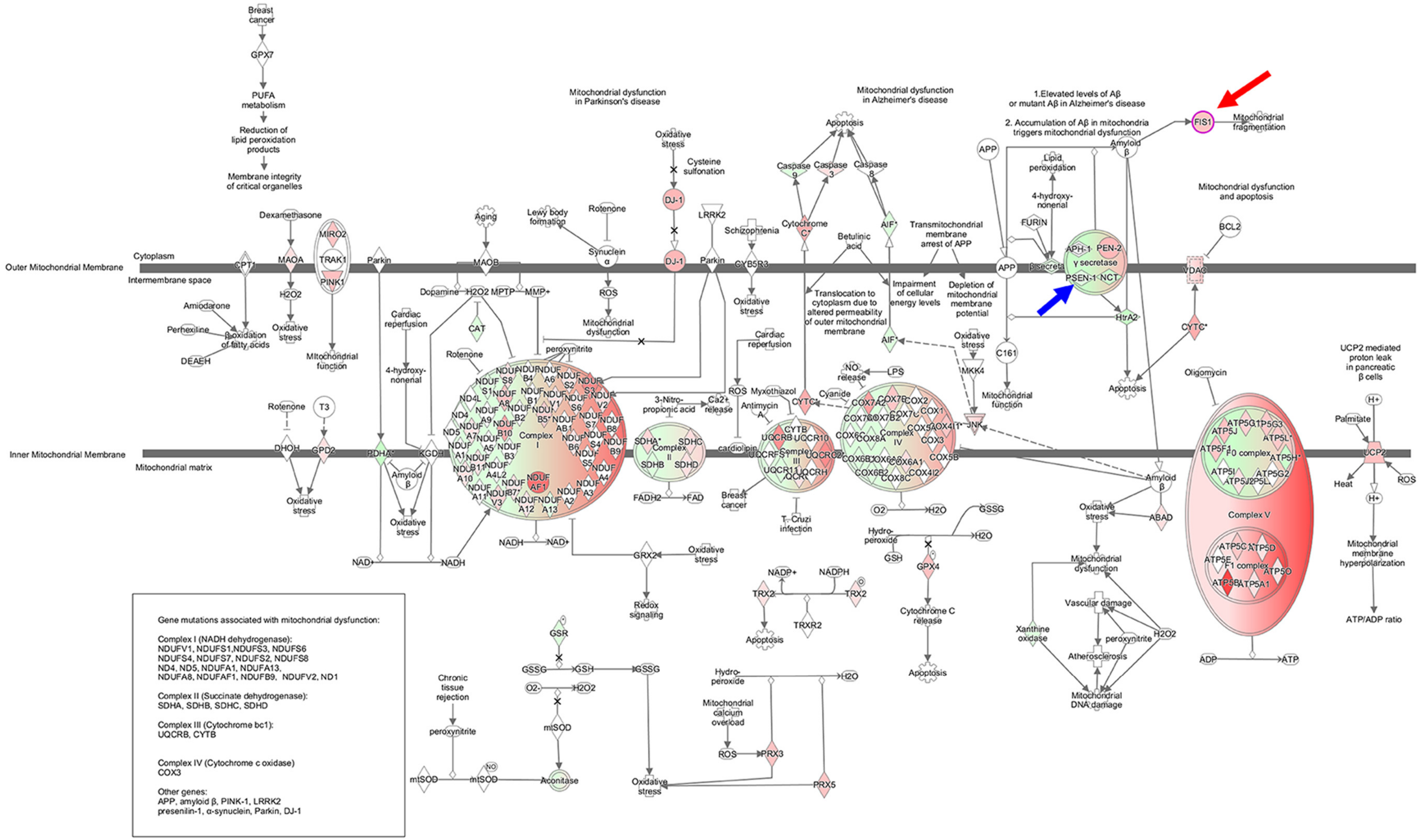

Figure S2. Transcriptional effect of Rlip deficiency on mitochondria. Brain RNA-Seq data from Rlip$^{+/-}$ mice (average of 5 males, 5 females, with technical replicates was overlaid on the canonical mitochondrial dysfunction pathway generated in Ingenuity Pathway Analysis. Red shaded nodes indicate increases and green shaded nodes indicate decreased expression in Rlip$^{+/-}$ mice, relative to wildtype. Overall, the figure shows that many mitochondrial function genes were aberrantly expressed in the brains of Rlip$_{+/-}$ mice. The arrows point out Fis1 (red), and presenilin-1 (PSEN1; blue).

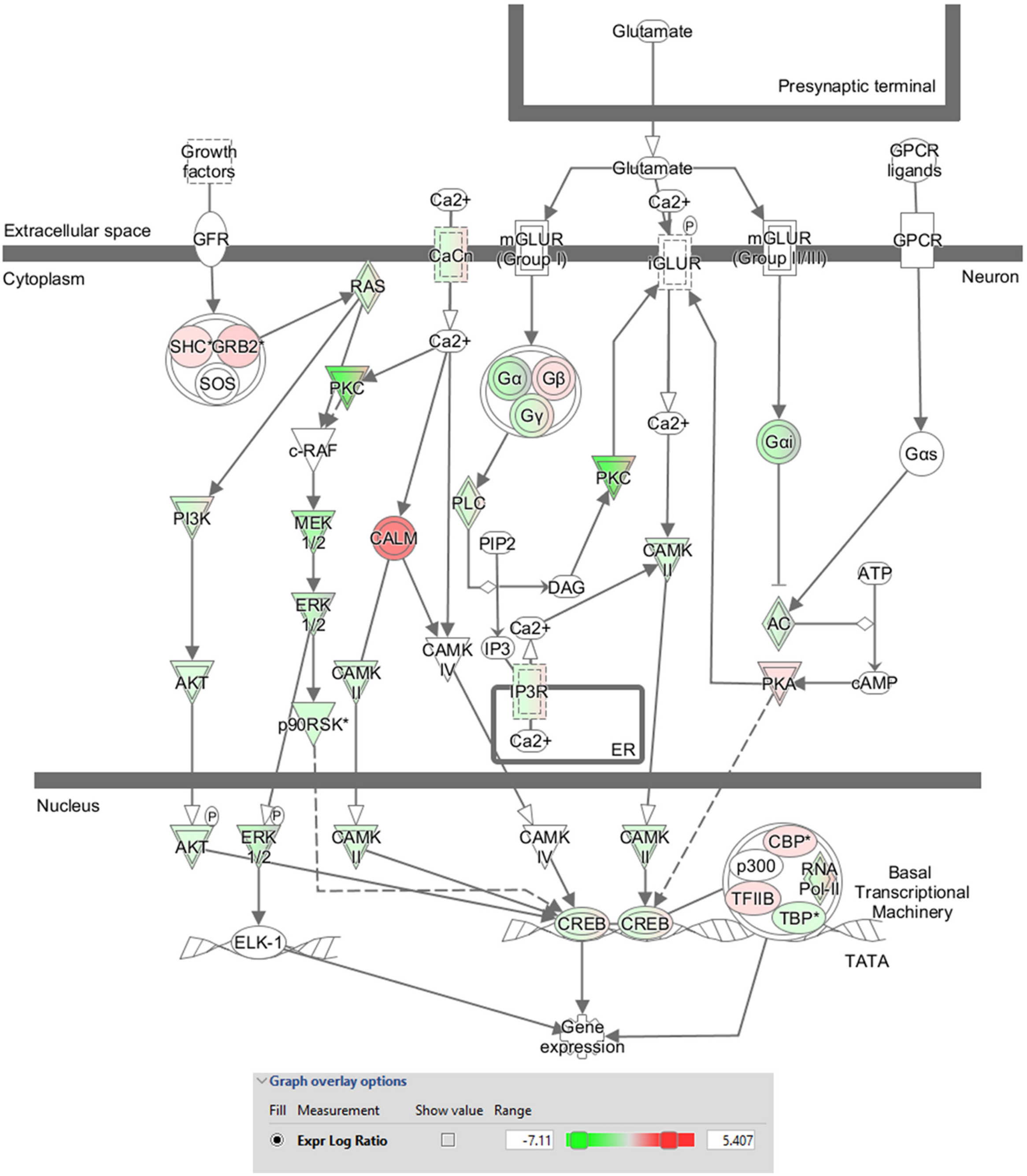

Figure S3. Transcriptomic effect of Rlip deficiency. Brain RNA-Seq data from Rlip$^{+/-}$ mice (average of 5 males, 5 females, with technical replicates) was overlaid on the canonical neuronal CREB signaling pathway generated in Ingenuity Pathway Analysis. Red shaded nodes indicate increases and green shaded nodes indicate decreased expression in Rlip$^{+/-}$ mice, relative to wildtype.
